# Pseudoaneurysm after arterial reconstruction in kidney transplant nephrectomy: A case report and literature review

**DOI:** 10.1002/iju5.12690

**Published:** 2024-01-23

**Authors:** Kuniaki Inoue, Shunta Hori, Mitsuru Tomizawa, Tatsuo Yoneda, Yasushi Nakai, Makito Miyake, Nobumichi Tanaka, Kiyohide Fujimoto

**Affiliations:** ^1^ Department of Urology Nara Medical University Kashihara Nara Japan; ^2^ Department of Prostate Brachytherapy Nara Medical University Kashihara Nara Japan

**Keywords:** kidney transplant nephrectomy, pseudoaneurysm, transplant kidney infection, vascular complication, vascular reconstruction

## Abstract

**Background:**

Pseudoaneurysm formation sometimes complicates transplant nephrectomy. We report a case of bleeding from a pseudoaneurysm after transplantation nephrectomy that resulted in shock and emergency endovascular treatment.

**Case presentation:**

A 56‐year‐old man underwent transplant nephrectomy 3 years and 9 months following transplantation for pyelonephritis‐related infection control. On postoperative day 7, he developed sudden pain in the lower abdomen and subsequently went into shock. A pseudoaneurysm at the anastomosis was detected, and urgent endovascular treatment was performed to stem the bleeding.

**Conclusion:**

Vascular complications, including pseudoaneurysms, following transplant nephrectomy can be life‐threatening, and comprehensive awareness is needed in careful postoperative management.

**Keynote message**

Kidney transplant nephrectomy with infection is associated with the risk of vascular complications and massive bleeding. Therefore, consideration of the vascular reconstruction method and being attentive to the risk of postoperative bleeding is important.

**Abbreviations & Acronyms**
ADPKD: autosomal dominant polycystic kidney disease
CRP: C‐reactive protein
CT: computed tomography
EIA: external iliac artery
GSR: graft survival rate
KT: kidney transplantation
MRSA: methicillin‐resistant *Staphylococcus aureus*
NA: not available
TK: transplant kidney
TN: transplant nephrectomy
VC: vascular complication

## Introduction

TN is commonly performed in patients with dysfunctional TKs with infection, pain, bleeding, graft malignancy, or anemia associated with inflammation. TN is sometimes complicated with life‐threatening pseudoaneurysms. Risk factors for VCs in TN have been reported to include infection, time to dialysis resumption, and chronic rejection.^1^, ^2^, ^3^ Here, we describe a patient who had a good immediate post‐TN course but who developed bleeding from a pseudoaneurysm when abdominal pressure was applied, resulting in shock and emergency endovascular treatment.

## Case presentation

**Fig. 1:**
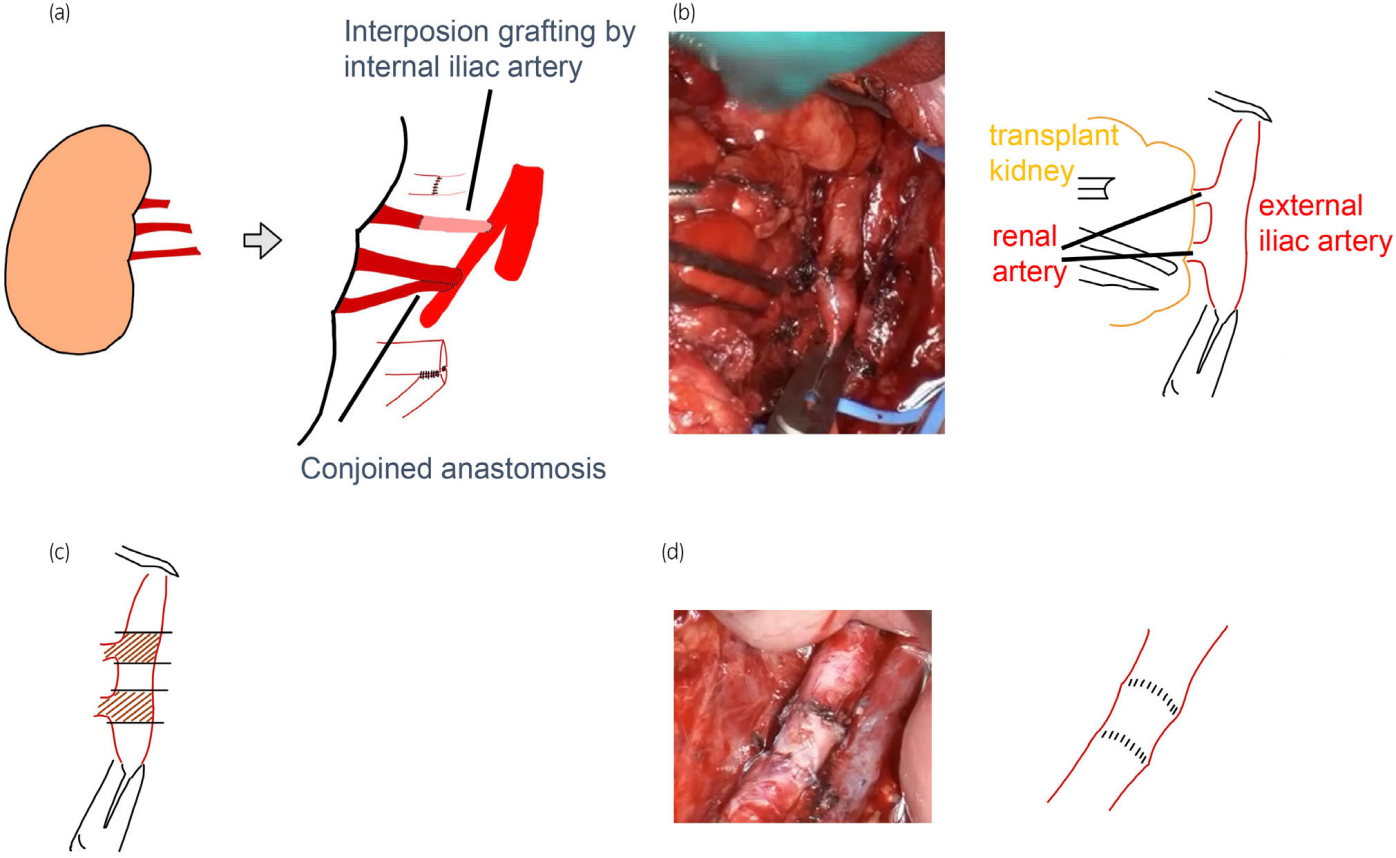
Illustration of operation techniques. (a) The transplanted kidney had three renal arteries, and the upper pole renal artery was short. The internal iliac artery was harvested as a graft and anastomosed end‐to‐end to the upper pole renal artery. Two main arteries were anastomosed and conjoined side‐to‐side. (b) The TK artery was very fragile and had severe adhesions to the surrounding area due to infection. Vascular suturing after severing the TK artery was extremely challenging. (c) The vessels were resected because the risk of VCs was high as the vessels had become fragile due to infection or other factors. (d) The uninfected EIA was used because of insufficient vessel length.

**Fig. 2:**
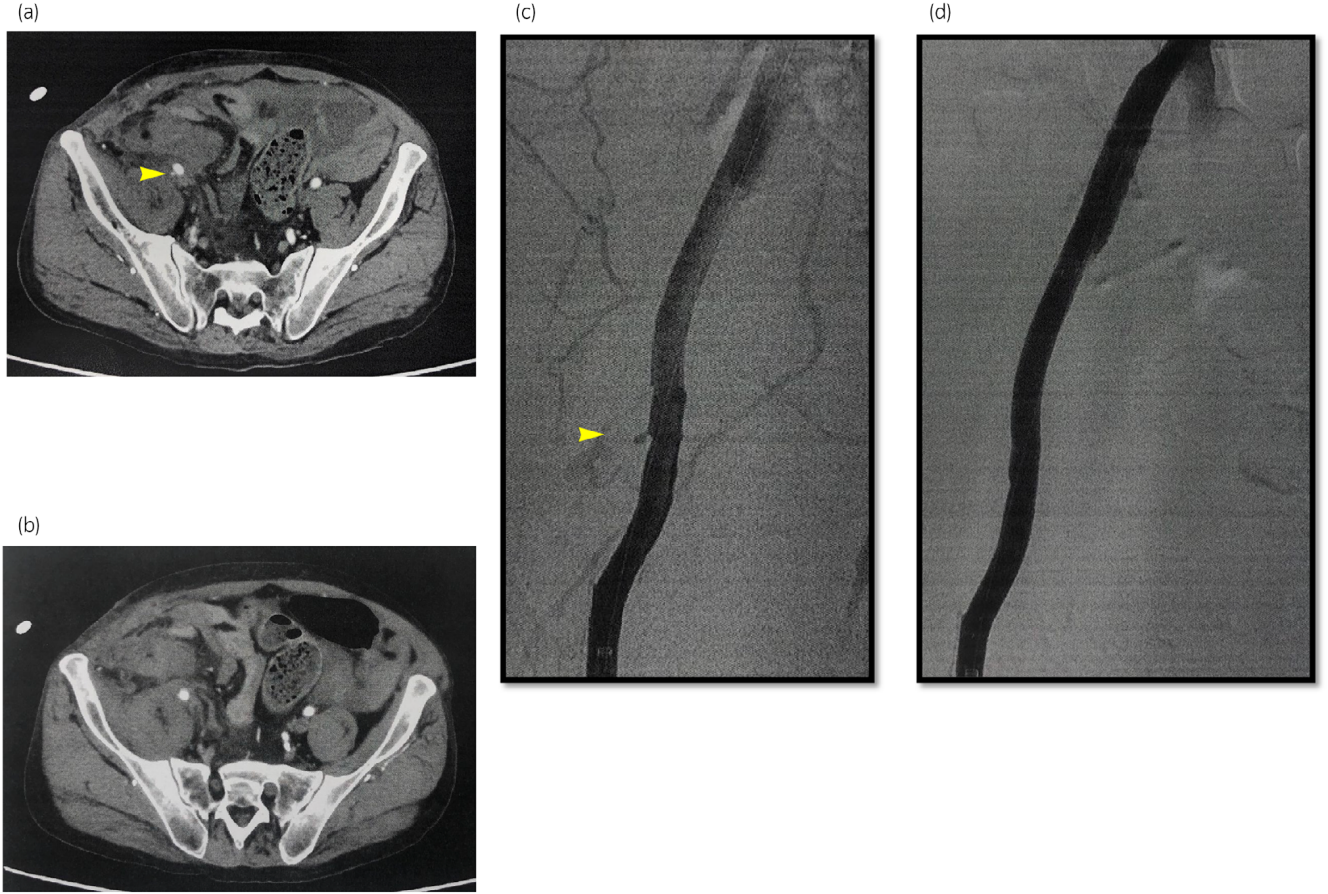
Contrast test to identify the bleeding site. (a) CT showing a pseudoaneurysm at the anastomosis of the EIA (b) The contrast material is also exposed to the abdominal cavity (c) Angiography showing the pseudoaneurysm (d) Covered stent placement (thickness, 8 mm; length, 10 cm).

A 52‐year‐old man who had undergone regular hemodialysis for 12 months for kidney failure secondary to ADPKD underwent ABO blood type‐incompatible living KT. The donor's three renal arteries were reconstructed to form two arteries, which were anastomosed with the recipient's EIA (Fig. 1a). Post‐KT, renal function did not improve, and a TK biopsy indicated acute tubular necrosis. Additional immunosuppressive drugs were administered, with minimal improvement. One month later, a nephrostomy tube was placed at the TK for stenosis at the uretero‐bladder anastomosis. At 10 months post‐KT, he developed recurrent pyelonephritis and was treated with antibiotics, but carbapenem‐resistant bacteria appeared among the causative organisms, and infection control was challenging. His kidney function continued to gradually deteriorate, and he was reintroduced to dialysis 17 months after KT. TN was performed 3 years and 9 months after KT because the patient requested re‐transplantation, and infection control was a priority. During surgery, the arteries around the transplanted kidney were resected as they appeared vulnerable owing to the effects of infection. The EIA was terminally anastomosed using a non‐infected artery (Fig. 1b–d). The central and distal sides of the EIA were sufficiently peeled off to perform tension‐free anastomoses. The patient was placed on bed rest until postoperative day 4 because of the high risk of postoperative VC. On postoperative day 7, when abdominal pressure was applied to defecate, the patient became aware of severe pain in the lower abdomen and subsequently went into shock. Emergency CT revealed a pseudoaneurysm at the anastomosis site and bleeding from the abdominal cavity into the pelvic cavity (Fig. 2a,b). Emergency endovascular treatment was performed, and the pseudoaneurysm disappeared after stenting (Fig. 2c,d). Postoperatively, red blood cell transfusion was performed until the hemoglobin levels stabilized. He was discharged 23 days after resection following CT confirmation that the hematoma had diminished in size and that there was no further source of infection. At 12 months post‐TN, our patient continued to progress without anemia or infection and was being prepared for re‐transplantation.

## Discussion

The British Transplantation Society reported indications for TN in 2014, and TN is used in many centers,^4^ with decisions on whether to perform TN based on perceived benefits and risks. The indications, advantages, and disadvantages of TN are listed in Table 1.^5^, ^6^

**Table 1:** Indications, advantages, and disadvantages of TN

Indications	Advantage	Disadvantage
Localizing symptoms (pain, infection, bleeding) that are resistant to medical therapy in a failed graft	Reduction in chronic rejection	Decreased GSR
To create space for re‐transplantation	Beneficial for survival on dialysis	Loss of residual kidney function
To enable complete withdrawal of immunosuppression	Prevention of graft intolerance syndrome	Difficulty in cross‐matching and longer waiting period for re‐transplant
Risk of graft rupture	Withdrawal of immunosuppressants	
Graft malignancy		Surgical complications
Refractory anemia with raised CRP		

TN‐related complications such as bleeding, hematoma, and infection have been reported, with incidence rates ranging from 5% to 48%.^7^ The incidence of VC has been reported to be 5.6%, with most reported cases being life‐threatening.^1^ Risk factors include infection of the anastomotic site,^1^ with the risk reported to be higher when TN is performed following 12 months of dialysis resumption.^2^ Chronic rejection may also be a risk factor for pseudoaneurysm.^3^ For our patient, underlying disease was also a risk factor for pseudoaneurysm, and patients with ADPKD are known to be prone to pseudoaneurysm during other common surgical procedures.^8^, ^9^, ^10^ Table 2 lists the relevant studies identified following a PubMed search relating to VC after TN. Pseudoaneurysms are common in VC, and the risk of mortality is high. Prophylactic methods for VC include immediate vascular reconstruction through bypassing artificial vessels or the saphenous vein at the time of TN, whereas non‐immediate vascular reconstruction involves ligation of the EIA, followed by antibiotic treatment and two‐phase bypass surgery.^1^ Eng *et al*. reported on two patients for whom artificial vessels or saphenous veins were used during TN. However, one patient died of MRSA wound infection and the other patient had a severe hemorrhage stemming from the site of the vein graft owing to MRSA infection.^1^ Immediate vascular reconstruction is associated with VCs in cases where reconstruction occurs in the presence of local infection. Non‐immediate vascular reconstruction is performed when local infection has resolved; therefore, VCs are unlikely to occur. According to this report, ligation of the EIA without reconstruction at the time of TN is not uniformly associated with limb ischemia.^1^ If the ipsilateral lower limb is deemed critically ischemic preoperatively, an extra‐anatomical bypass, such as a femoral‐femoral bypass graft employing antibiotics, is the preferred reconstruction method. However, this report appears not to have been updated as a search of more recent literature did not identify any reports on ligation of the EIA. Ligation of the EIA was not a treatment option at our hospital because of the risk of lower limb ischemia.

**Table 2:** Indications, advantages and disadvantages of TN

Author (references no.)	Patient no.	Indication for TN	VC	Interval between TN and diagnosis of VC	Clinical presentation	Treatment	Out come (post TN days)
Payne *et al*.^11^	1	Haemorrhage	EIA pseudoaneurysm post‐TN	1 month	Wound haemorrhage	EIA ligation	Death (6 months)
Payne *et al*.^11^	2	Early graft failure	EIA pseudoaneurysm post‐TN	6 weeks	Wound haemorrhage	EIA ligation	Death (1 month)
Payne *et al*.^11^	3	Acute rejection, sepsis	Fistula from iliac artery to appendix	2 months	Intestinal haemorrhage	EIA ligation, resection of the appendix	Death
Brown *et al*.^12^	4	Acute rejection, sepsis	EIA bleeding post‐TN	2 weeks	Wound haemorrhage	EIA ligation	Alive
Brown *et al*.^12^	5	Acute rejection	EIA bleeding post‐TN	1 week	Abdominal pain, pulsatile mass	EIA ligation	Alive
Eng *et al*.^1^	6	Haemorrhage, sepsis	EIA pseudoaneurysm post‐TN	NA	NA	EIA ligation femoral‐femoral cross over grafting	Death (50 days)
Eng *et al*.^1^	7	Chronic rejection	EIA injury at TN	NA	NA	EIA ligation	Alive
Eng *et al*.^1^	8	Renal vein thrombosis, sepsis	EIA pseudoaneurysm and bleeding at TN	0 days	NA	EIA ligation	Alive
Eng *et al*.^1^	9	Acute rejection, renal vein thrombosis	EIA injury at TN bleeding post‐TN	21 days	NA	EIA ligation, saphenous vein grafting	Alive
Eng *et al*.^1^	10	Acute rejection	EIA bleeding post‐TN	NA	NA	EIA ligation femoral‐femoral cross over grafting	Alive
Eng *et al*.^1^	11	Chronic rejection	EIA injury at TN	NA	NA	EIA ligation	Death (3 days)
Eng *et al*.^1^	12	Haemorrhage, sepsis	EIA bleeding at TN	NA	NA	EIA ligation	Death (3 months)
Eng *et al*.^1^	13	Acute rejection, sepsis	EIA pseudoaneurysm post‐TN	7 weeks	NA	Stent grafting	Alive
Eng *et al*.^1^	14	Chronic rejection	EIA pseudoaneurysm post‐TN	6 months	NA	Stent grafting	Alive
Bracale *et al*.^3^	15	Chronic rejection	EIA pseudoaneurysm post‐TN	37 months	Abdominal pain, pulsatile mass	EIA ligation, interposition grafting	Death (75 months)
Bracale *et al*.^3^	16	Renal vein thrombosis	EIA pseudoaneurysm post‐TN	7 months	Asymptomatic	EIA ligation, interposition grafting	Alive
Bracale *et al*.^3^	17	Acute rejection	EIA pseudoaneurysm post‐TN	71 days	Local discomfort, pulsatile mass	Stent grafting	Alive
Bracale *et al*.^3^	18	Renal arterial thrombosis	EIA pseudoaneurysm post‐TN	6 months	Abdominal pain, anemia, fever, tender mass	EIA ligation, interposition grafting	Alive
Bracale *et al*.^3^	19	Chronic rejection	EIA pseudoaneurysm post‐TN	5 months	Local pain, pulsatile mass	Stent grafting	Alive
Bracale *et al*.^3^	20	Acute rejection, sepsis	EIA pseudoaneurysm post‐TN	13 days	Abdominal pain, fever, hypotension	EIA ligation femoral‐femoral cross over grafting	Alive
Siddiqui *et al*.^13^	21	Acute rejection, renal vein thrombosis	EIA bleeding post‐TN	5 days	Abdominal pain	Stent grafting	Alive
Bracale *et al*.^14^	22	Early graft failure	EIA pseudoaneurysm post‐TN	3 months	Abdominal pain, severe dyspnea, tachycardia	Stent grafting	Alive
Borges *et al*.^15^	23	Chronic rejection, sepsis	EIA pseudoaneurysm post‐TN	1 year	Abdominal pain, pulsatile mass	Resection of the pseudoaneurysm and anastomosis between the common iliac artery and the Carrell patch	Alive

In addition, care must be taken postoperatively as bleeding can occur a week after surgery, as in this case. A sufficient period of postoperative rest and thorough evaluation using imaging studies are necessary before discharge from the hospital.

## Author contributions

Kuniaki Inoue: Writing – original draft. Shunta Hori: Conceptualization. Mitsuru Tomizawa: Conceptualization; writing – review and editing. Tatsuo Yoneda: Conceptualization; writing – review and editing. Yasushi Nakai: Conceptualization; writing – review and editing. Makito Miyake: Conceptualization; writing – review and editing. Nobumichi Tanaka: Project administration. Kiyohide Fujimoto: Project administration; supervision.

## Conflict of interest

The authors declare no conflict of interest.

## Approval of the research protocol by an Institutional Reviewer Board

Not applicable.

## Informed consent

Consent to participate and for publication were acquired from the patient.

## Registry and the Registration No. of the study/trial

Not applicable.
